# Ecological divergence of syntopic marine bacterial species is shaped by gene content and expression

**DOI:** 10.1038/s41396-023-01390-4

**Published:** 2023-03-04

**Authors:** Brent Nowinski, Xiaoyuan Feng, Christina M. Preston, James M. Birch, Haiwei Luo, William B. Whitman, Mary Ann Moran

**Affiliations:** 1 Department of Marine Sciences, University of Georgia, Athens, GA 30602 USA; 2 Simon F. S. Li Marine Science Laboratory, School of Life Sciences and State Key Laboratory of Agrobiotechnology, The Chinese University of Hong Kong, Shatin, Hong Kong SAR; 3 Monterey Bay Aquarium Research Institute, Moss Landing, CA 95039 USA; 4 Department of Microbiology, University of Georgia, Athens, GA 30602 USA

**Keywords:** Microbial ecology, Microbial ecology

## Abstract

Identifying mechanisms by which bacterial species evolve and maintain genomic diversity is particularly challenging for the uncultured lineages that dominate the surface ocean. A longitudinal analysis of bacterial genes, genomes, and transcripts during a coastal phytoplankton bloom revealed two co-occurring, highly related *Rhodobacteraceae* species from the deeply branching and uncultured NAC11-7 lineage. These have identical 16S rRNA gene amplicon sequences, yet their genome contents assembled from metagenomes and single cells indicate species-level divergence. Moreover, shifts in relative dominance of the species during dynamic bloom conditions over 7 weeks confirmed the syntopic species’ divergent responses to the same microenvironment at the same time. Genes unique to each species and genes shared but divergent in per-cell inventories of mRNAs accounted for 5% of the species’ pangenome content. These analyses uncover physiological and ecological features that differentiate the species, including capacities for organic carbon utilization, attributes of the cell surface, metal requirements, and vitamin biosynthesis. Such insights into the coexistence of highly related and ecologically similar bacterial species in their shared natural habitat are rare.

## Introduction

Environmental specialization has been proposed as an important mechanism for genetic diversification of bacterial populations into ecologically distinct species-level lineages [1, 2]. Genetic transitions in marine bacteria (such as SAR11 [2], *Vibrio splendidus* [3], and *Prochlorococcus* [4]) have been attributed to adaptation to environmental factors that include light, temperature, and nutrient or organic matter availability. These factors can operate at biogeographic scales, such as at the dimension of global ocean currents [2], as well as at micron-scales, such as on individual particle surfaces [3]. However, in the situation of syntopic bacterial speciation, defined as a special case of sympatry in which closely related species occupy the same microenvironment at the same time [5], no physicochemical barriers to gene flow are present. Species differentiation in this case is thought to be facilitated when adaptation at only a few loci is sufficient for genetic isolation [6].

During a phytoplankton bloom in Monterey Bay, CA, USA in the fall of 2016 [7], the in situ robotic Environmental Sample Processor (ESP) [8] collected and stored microbial cells for nucleic acid analysis at Station M0. 16S rRNA gene amplicon sequencing data from 41 dates during this longitudinal study [9] revealed that the most abundant taxon in the bacterial community was represented by an amplicon sequence variant (ASV) belonging to a marine Roseobacter (*Rhodobacteraceae*) from the NAC11-7 lineage. Roseobacter species vary dramatically in life history characteristics, some with large genomes (>4 Mb) that are amenable to culturing, and others with streamlined genomes (<2.3 Mb) that largely remain uncultured [10]. The abundant Monterey Bay taxon belonged to the latter. Protein sequences mapping to genomes represented by this ASV indicated high relatedness to the only previously cultured member of the streamlined NAC11-7 lineage, strain HTCC2255, which was isolated from seawater by dilution to extinction [11] but lost subsequently from culture. Consequently, members of the lineage remain poorly studied despite their ubiquity in surface seawater environments [12–15].

Streamlined members of the marine Roseobacter group typically exhibit low sequence divergence in their 16S rRNA genes [16–19]. Analysis of Monterey Bay bloom metagenomic and single-cell data in fact suggested that the NAC11-7 16S rRNA gene amplicon actually represented two sequence-discrete genome clusters that can be classified as syntopic species. Here, we use longitudinal sequencing of bacterial metagenomes during the bloom to confirm the presence of syntopic NAC11-7 species. Comparative analysis of genome content and in situ per cell transcript inventories over 7 weeks of sampling provided specific insights into the diverged physiological and ecological roles of these species in their natural environment.

## Methods

### Sample collection

Microbial cells in the ≤5 μm to ≥0.22 μm size range were collected at Monterey Bay Station M0 over a 52 d period from September 26 through November 16, 2016, using either a moored autonomous robotic instrument (the Environmental Sample Processor; ESP) with daily sampling, or when the ESP was offline from October 8 to October 31, using Niskin bottles deployed from a boat with sampling every 2-3 days. Seawater was sampled at ~6 m depth, and at 10:00 AM Pacific Daylight Time to normalize for diel effects on microbial gene expression [20, 21]. From this sampling, 88 16S rRNA gene amplicon libraries, 84 metagenomes, and 47 transcriptomes (*n* = 2–4) were generated. Additional metagenomes and single-cell genomes (SAGs) from a Fall 2014 Monterey Bay study [22] were included in the analysis.

### Sequencing and assembly

DNA and RNA sequencing and assembly of libraries representing the microbial community were carried out as described previously [9, 23, 24]. Our protocol included addition of two genomic DNA internal standards from non-marine bacteria *Thermus thermophilus* and *Blautia producta* [25, 26] and two mRNA internal standards (each ~1000 bp) transcribed from custom templates [27]; these were added in known amounts to sample filters prior to processing. For each metagenomic sample assembly, reads from all metagenomic samples were mapped to contigs to generate coverage patterns through time (Bowtie2 v2.3.4.1) [23, 28]. The contigs were binned using MetaBAT 2.12.1 [29], using “jgi_summarize_bam_contig_depths” to incorporate coverage patterns across samples and the “metabat2” function to generate metagenome-assembled genome (MAG) bins for each sample. All resulting genome bins were dereplicated using dRep 2.3.0 [30] at a 95% Average Nucleotide Identity (ANI) threshold, resulting in 81 high-quality MAGs based on CheckM v1.0.12 [31] that were ≥75% complete, including MAGs from the internal standard genomes. Two of the high-quality MAGs were members of the NAC11-7 lineage. Three single-cell genomes (SAGs) also representing NAC11-7 bacteria were acquired from surface seawater from a 2014 Monterey Bay study [22] as described previously. An additional 25 NAC11-7 SAGs were acquired from this 2016 study, and these were processed and sequenced through the Joint Genome Institute single-cell genomics pipeline [28]. ANI between the NAC11-7 genomes was calculated using the ANI/AAI-Matrix program as part of the enveomics collection toolbox [32].

### Identifying genomic differences

To obtain the pangenome of the two NAC11-7 lineage species, metagenomic and metatranscriptomic reads from Monterey Bay from 2016 [9] were mapped to the 31 NAC11-7 genomes (the original HTCC2255 isolate [33] along with 2 MAGs and 28 SAGs from this study) using Bowtie2. anvi’o v6.1 [34] was used to create a database of DNA, amino acid sequences, and read mapping profiles of the 31 genomes. Genes were annotated using three databases: ‘anvi-run-ncbi-cogs’ with the December 2014 release of the Clusters of Orthologous Groups (COGs) database [35], EggNOG-mapper v1.0.3 with the EggNOG v4.5.1 database [36], and KofamKOALA database v. 2019-03-20 [37] using blastp with E-value cutoffs of 1 × 10^−5^ and identities ≥ 30%. Protein-encoding genes were clustered based on sequence homology using the program ‘anvi-pan-genome’ with parameters ‘--use-ncbi-blast’, ‘--minbit 0.5’, and ‘--mcl-inflation 10’. The pangenome was visualized using the program ‘anvi-display-pan’.

This gene clustering approach was used to initially distinguish potentially unique genes (found in one species) from core genes (shared by most members of both species). Since the incomplete MAG and SAG genomes could lead to erroneous assignment of genes as unique, however, we mapped metagenomic reads to each putative unique gene to obtain the percent identity distribution of the mapped reads. Specifically, BBmap v38.73 [38] was used with the bbmap.sh script and parameter “idhist” with a minimum alignment identity cutoff of 60%. From the resulting histograms, putative unique genes for which some mapped reads fell within the 70–90% identity range were flagged for manual analysis to determine whether a peak indicative of a second species was evident. After manual inspection, those determined to have a bimodal pattern were reassigned to the core gene category; all genes retained as unique had ratios >2:1 of reads mapping at >95% to reads mapping at 70–90%, and no second species peak.

Genes identified as unique in this analysis were queried for evidence of acquisition by lateral gene transfer (LGT) using analysis of BLAST hits to NCBI RefSeq v. 214 or, for the HTCC2255 isolate genome, using the IMG/MER pipeline. Genes with best hits outside the *Rhodobacteraceae* lineage and having few or lower-scoring hits within were considered candidates for horizontal transfer. For this analysis, best BLAST hits were defined as those with bit scores within 90% of the highest bit score for that gene [39]. Genes potentially acquired by allelic replacement from closely related lineages were identified by synonymous substitution rate (*d*_*S*_) clustering [40]. A *d*_*S*_ matrix was generated of pairwise comparisons across all genomes of single-copy core genes, with MAG genomes excluded due to potential chimeric segments. Synonymous substitutions are largely neutral and within-species *d*_*S*_ values mostly comparable. Outlier genes with unusually large *d*_*S*_ values were analyzed for discrepancies between gene tree topology and species tree topology [40]. Missing values were imputed using the R package ‘ClustImpute’ [41] and the *d*_*S*_ outlier genes were identified by the K-means clustering method.

### Identifying differentially expressed genes

A spike of known copy numbers of internal mRNA and genomic DNA standards at the initiation of sample preparation enabled calculation of transcripts per gene copy by sample date [25, 42]; specifically, we calculated the average number of mRNAs for a given gene in cells of each species in the same water sample [25]. Gene expression ratios were calculated by dividing the internal standard-normalized transcripts per liter of seawater by internal standard-normalized genes per liter. The metatranscriptome samples collected manually when the autonomous ESP sampler was offline (for 3 weeks in the 7.5 week sampling period) had considerably lower per-gene mRNA copy numbers; therefore only samples collected autonomously by the ESP were used in comparative expression analysis. The lower mRNA levels in manual samples were attributed to the 1.5 h gap between manual collection at Station M0 and fixation in the laboratory. By contrast, ESP samples were fixed inside the instrument immediately after filtration by flooding with preservative, minimizing degradation.

### Species stoichiometry in coastal marine environments

Calculation of the ratio of MBARI-HTCC2255:MBARI-C16 in the Tara Oceans [43], Galapagos [44], and 2014 Monterey Bay metagenomic datasets [22] was carried out by blastn alignment to six genes distinguishing the species: two highly prevalent core genes and the two most prevalent genes to each species. Blastn parameters used to retain reads for abundance counts were an E-value cutoff of 1 × 10^−5^, query coverage ≥ 80%, and identity ≥95%.

## Results and discussion

### Abundant syntopic Roseobacter species

**Fig. 1:**
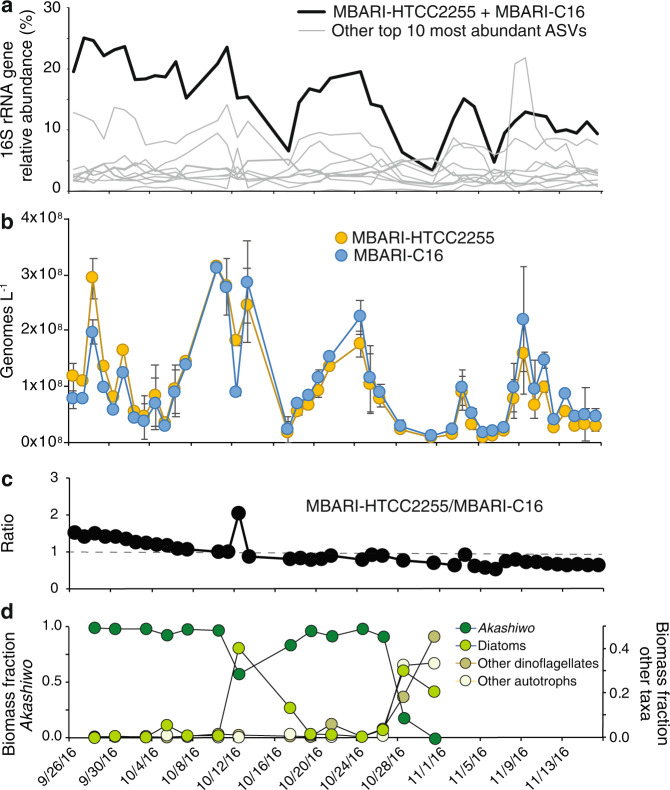
Abundance of NAC11-7 syntopic species. **a** Relative abundance of the top 10 most abundant ASVs; the bolded line represents the ASV that is identical between MBARI-HTCC2255 and MBARI-C16, while the gray lines represent the nine other most abundant ASVs. **b** Copies per liter of seawater of each species’ genome. **c** Ratio of MBARI-HTCC2255 to MBARI-C16 genomes. **d** Phytoplankton biomass composition (September 28- through October 31, 2016). *Akashiwo sanguinea* was the dominant phytoplankton species through the majority of the bloom.

The surface bacterioplankton community of Monterey Bay in the Fall of 2016 was dominated by a 16S rRNA gene amplicon sequence variant (ASV) that averaged 15% of community sequences, twice that of the next most abundant ASV (Fig. 1a). This ASV aligned with 100% identity to the V4 region of the 16S rRNA gene sequence of Roseobacter bacterium HTCC2255, represented in previous sequence libraries from Monterey Bay [15, 21, 45], Puget Sound, and the North Sea [46, 47], among other locations [48]. The streamlined NAC11-7 lineage to which HTCC2255 belongs is most abundant in coastal areas and often linked to phytoplankton blooms [16, 19, 45, 49, 50], as was the case during the Monterey Bay sampling [7]. As a basal member of the marine *Rhodobacteraceae* [51, 52], HTCC2255 has distinct characteristics that distinguish it from the readily cultured members of this clade, including one of the smallest genomes (2,209 genes) and one of the lowest %G+C contents (36.7%). Loss of the original HTCC2255 isolate from culture and lack of other NAC11-7 lineage isolates has made studies of the ecology and evolution of this taxon challenging. The high abundance of HTCC2255-like sequences over 7 weeks of a natural phytoplankton bloom provided us with a unique opportunity to study the population structure and ecology of a streamlined lineage in situ.

**Fig. 2:**
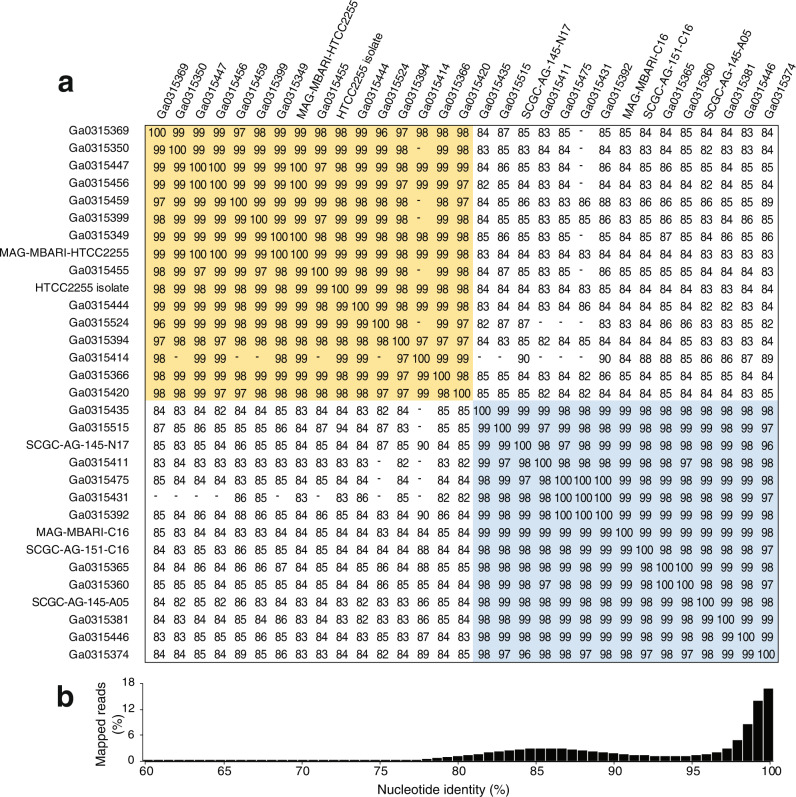
Evidence for NAC11-7 lineage sequence-discrete clusters represented by a single ASV in Monterey Bay. **a** Estimated all-vs-all Average Nucleotide Identity (ANI) distances and similarity clustering of 31 genomes from assembled metagenomic data (MAGs), single amplified genomes (SAGs), and the HTCC2255 isolate. Values are missing if the number of shared genes was too low for accurate ANI calculations. Gold shading indicates MBARI-HTCC2255 clade genomes, all clustering at >95% ANI, and blue shading indicates MBARI-C16 clade genomes, all clustering at >95% ANI. **b** Nucleotide percent identity of metagenomic reads mapping to the HTCC2255 isolate genome (example from metagenomic sample 46D).

Eighty-four metagenomes were generated over the study, from which MAGs were assembled from each library independently and then binned by nucleotide composition and read recruitment patterns across libraries, followed by dereplication at 95% to obtain a high-quality MAG for each abundant species in the bloom. Genomic DNA from bacteria serving as internal standards provided a test of our assembly and binning methods, as MAGs identical to each reference genome were recovered [average nucleotide identity (ANI) [32] calculations of 100% for both *T. thermophilus* and *B. producta*]. Two high-quality NAC11-7 lineage MAGs related to the HTCC2255 isolate genome were present. One was nearly identical to HTCC2255 (>99%) (designated MAG-MBARI-HTCC2255) (Fig. 2a), while the other was only 84% identical to HTCC2255 (designated MAG-MBARI-C16) (Fig. 2a).

Twenty-five single-amplified genomes (SAGs) were also obtained from the bloom bacterial community that clustered with either MAG-MBARI-HTCC2255 (14 SAGs with >95% ANI) or MAG-MBARI-C16 (11 SAGs with >95% ANI) (Fig. 2a). Three additional SAGs retrieved two years earlier in Fall 2014 at the same location [22] (Table S1) had >95% ANI to MAG-MBARI-C16. Using this expanded number of genomes (31 total; 2 best-quality MAGs, 28 SAGs, and the HTCC2255 isolate genome) (Fig. S1), pairwise ANI comparisons indicate that the two groups had within- and between-cluster relatedness consistent with an ANI-based species delineation [53] (Fig. 2a), hereafter designated MBARI-HTCC2255 and MBARI-C16. All SAGs that yielded a 16S rRNA gene from assembly, regardless of species delineation, shared identical V4 regions with each other and the original HTCC2255 isolate, and between species full length 16S rRNA gene identity was 99.9%.

Defining bacterial species is challenging, and indeed whether and how bacteria differentiate into identifiable species has been debated [54]. Current species definitions can be based on the degree of dissociation of DNA extracted from bacterial isolates [55]; the identification of ecotypes occupying the same niche and purged by natural selection [56]; the phylogenetic clustering of single-copy marker genes (such as ribosomal proteins and t-RNA synthetases) [57, 58]; and discontinuities in whole-genome similarity measures [53, 59]. For analysis of these two uncultured Roseobacter clusters, we used the whole-genome similarity measure of ANI, which tallies the nucleotide identity of all orthologous genes shared between two genomes [53]. Previous pairwise analysis of nucleotide identity across known bacterial species has revealed that ANI values for populations classified into the same species with traditional methods are typically ≥95%; those for populations classified into different species are typically ≤83%; and few identity values fall into the gap in between [53]. This gap in ANI identity is proposed to begin at the approximate barrier to genetic exchange, below which populations are freed to diverge independently [1]. For the HTCC2255 and MBARI-C16 groups, their 84% ANI (Fig. 2a) identifies them as distinct species with a high level of genomic similarity that is rare for bacterial species in the same environment at the same time [54].

We recognized the possibility that bacterial populations with intermediate relatedness could indeed be present but their sequences not captured in the SAGs or MAGs. To rule this out, unassembled Monterey Bay metagenomic reads were mapped to the HTCC2255 isolate genome. A bimodal pattern emerged in which mapped reads binned into a peak at 100% nucleotide identity (44% of mapped reads) or a peak at 85% identity (41%) (Fig. 2b) consistent with two highly related but sequence discrete taxa. Mapping instead to the MAG-MBARI-C16 genome resulted in the same bimodal pattern. Thus the possibility of abundant intermediate populations was ruled out, and the closely related clusters existing side-by-side in a natural environment established HTCC2255 and MBARI-C16 as syntopic species [5].

### Overview of genes and transcripts

Analysis of the 84 Monterey Bay metagenomic datasets (*n* = 2 or 3 for 35 sample dates, *n* = 1 for 6) generated 19,520 open reading frames encoding 9,937 genes from MBARI-HTCC2255 cluster genomes and 9583 from MBARI-C16 genomes. Reads from the unassembled metagenomic datasets were then mapped to this gene set, and the recovery ratio of the internal standards in each sample (exogenous *T. thermophilus* and *B. producta* DNA) was applied to the coverage of the protein-encoding genes [25, 26] to calculate abundance. Average abundances for MBARI-HTCC2255 and -C16 were 9.4 × 10^7^ and 9.6 × 10^7^ genomes L^−1^, respectively, and the average ratio of abundances (MBARI-HTCC2255:MBARI-C16) was 0.85 ± 0.36 (Fig. 1b).

Unique (i.e., species-specific) genes were identified by pangenomic clustering [60] of protein-encoding genes from the 31 genomes. This identified 2,872 gene clusters: 577 as singletons (found only in one genome) that were not considered further; 2,081 as core genes present in both species (Figs. S1, S2); and 214 as candidate unique genes, i.e., found multiple times but only in one species. Since incomplete MAG and SAG genome sequences could artificially inflate designations of unique genes, the unassembled metagenomic reads were mapped to the candidate unique gene clusters. For 115 gene clusters, reads mapped in two identity peaks (at 100% and 84%, as in Fig. 2b and Fig. S2) indicating a shared gene. These were re-classified as core to bring the total core genes to 2,196 genes. The final tally of unique genes was 59 genes from MBARI-HTCC2255 genomes and 40 from MBARI-C16 genomes (Tables S2, S3).

Analysis of the 47 Monterey Bay metatranscriptomes (*n* = 1, 2, or 3 for 28 sample dates) similarly utilized internal standards consisting of known amounts of artificial mRNAs added at the initiation of sample preparation. Specifically, we calculated the average number of mRNAs for a given gene in a cell of each species in the same environment. Bacterial cells typically maintain a low inventory of mRNA relative to genes, and considering all genes and sample dates, the NAC11-7 cells averaged 0.07 transcripts per gene copy. This value is 6-fold lower than for *E. coli* cells growing exponentially under ideal laboratory conditions [~0.4 transcripts per gene copy [61, 62]], as expected given growth rate differences. Moreover, it matches inventories reported for marine bacterial communities [~0.07 transcripts per gene copy, based on an assumption of ~200 mRNAs cell^−1^ [42] and 2,900 genes genome^−1^ [63]]. The most highly expressed NAC11-7 genes had up to 4.9 transcripts per gene copy, however (Table S4).

**Fig. 3:**
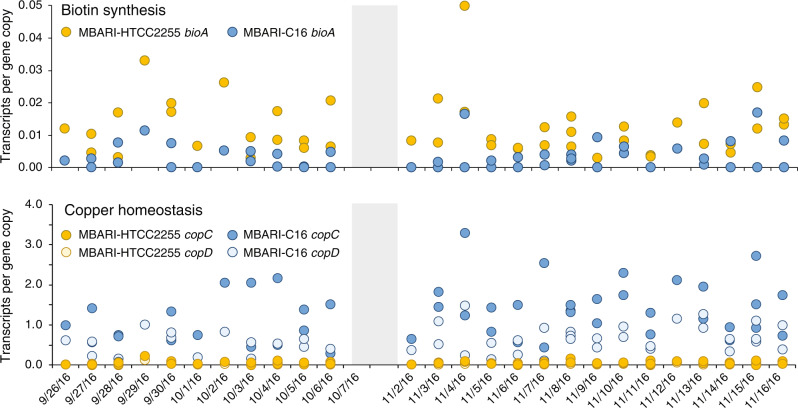
Example core genes with significant differences in expression levels (transcripts per gene copy) during the Fall 2016 Monterey Bay bloom. Gray shading indicates the time period when autonomous sample collection by the Environmental Sample Processor was not available.

Upregulated genes were identified based on significant differences between the species in per-gene transcript inventories when analyzed across the full sample set (T-Test; FDR adjusted *p* ≤ 0.05 and fold-difference ≥2). Fifty-one of the 2196 core genes were upregulated in one of the species under the Monterey Bay bloom conditions (Table 1, S5). Evidence that the upregulation signal was not due to stochastic events came from two sources: differentially expressed genes were often located in genomic regions that also had unique genes, laterally transferred genes, and/or *dS* outliers (detailed below); and patterns of differential expression were consistent across multiple sequential sample dates (Fig. 3).

**Table 1:** Core gene clusters with ≥2-fold expression differences between species (MBARI-HTCC2255/ MBARI-C16) in MBARI-HTCC2255 genomes (16 genes) and MBARI-C16 genomes (35 genes), organized by functional assignment.

Gene Cluster ID	MBARI Clade	Representative IMG Gene ID	Transcripts per Gene Copy	Functional Assignment	Annotation
GC_00000821	HTCC2255	2517702172	2.22	cell surface	lipoprotein
GC_00001797	HTCC2255	2517701166	4.03	cofactor	adenosylmethionine-8-amino-7-oxononanoate transaminase (*bioA*)
GC_00002130*	HTCC2255	2517702682	2.64	detoxification/stress	LamB YcsF family protein
GC_00001582	HTCC2255	2517701925	2.17	DNA/RNA related	ribosome-binding factor A
GC_00002206	HTCC2255	Ga0315366_1131	3.21	electron transfer	cytochrome B561
GC_00002263	HTCC2255	Ga0315446_1343	2.05	metabolism	alcohol dehydrogenase
GC_00001518	HTCC2255	2517702423	2.10	metabolism	acetyltransferase GNAT family
GC_00001461	HTCC2255	2517702566	2.06	metabolism (choline)	sarcosine oxidase alpha subunit
GC_00001635	HTCC2255	2517701161	2.79	metabolism	oxidoreductase
GC_00000705	HTCC2255	2517701188	2.02	metabolism (2^o^ met)	2og-fe(ii) oxygenase
GC_00002277	HTCC2255	2517701018	2.32	metabolism (sugar)	glycosyl transferase, family 25
GC_00000600	HTCC2255	2517702623	2.40	regulation	response regulator
GC_00002104	HTCC2255	2517702580	3.08	transport	transporter, RhaT family, DMT superfamily
GC_00001120	HTCC2255	2517701563	2.02	transport	phosphate transporter
GC_00000895	HTCC2255	2517701818	2.00	unknown/other	thioesterase
GC_00000814	HTCC2255	2517702318	2.08	unknown/other	cupin protein
GC_00000115	MBARI-C16	2517702112	0.43	cell cycle	Maf-like septum formation protein
GC_00002052§	MBARI-C16	2517701230	0.19	cell surface	spore coat protein U domain (pilin)
GC_00002223§	MBARI-C16	n/a	0.25	cell surface	spore coat protein U domain (pilin)
GC_00000585	MBARI-C16	2517701653	0.44	cell surface	SmpA OmlA lipoprotein
GC_00001895	MBARI-C16	2517702558	0.42	cofactor	CoA-binding domain protein
GC_00002185	MBARI-C16	3300032478	0.46	cofactor	dihydrolipoyl dehydrogenase
GC_00001331	MBARI-C16	2517701226	0.05	detoxification/stress	copper binding, *copC*
GC_00001317	MBARI-C16	2517701227	0.06	detoxification/stress	copper resistance, *copD*
GC_00001072	MBARI-C16	2517702209	0.33	detoxification/stress	NTP pyrophosphohydrolases
GC_00000844	MBARI-C16	2517702103	0.47	detoxification/stress	ATP-dependent HslUV protease
GC_00001148	MBARI-C16	2517702914	0.49	detoxification/stress	stress responsive alpha-beta barrel domain
GC_00001832	MBARI-C16	2517700903	0.49	DNA/RNA related	deoxyribodipyrimidine photo-lyase
GC_00000902	MBARI-C16	2517701225	0.15	electron transfer	cytochrome C family protein
GC_00001837	MBARI-C16	2517701218	0.33	electron transfer	cytochrome
GC_00001790	MBARI-C16	2517702559	0.34	electron transfer	ferredoxin
GC_00001063	MBARI-C16	2517701726	0.50	electron transfer	potential cyctchrome biogenesis
GC_00001625	MBARI-C16	2517702800	0.18	metabolism (amino acid)	decarboxylase
GC_00002149	MBARI-C16	2517701746	0.47	metabolism (amino acid)	deiminase
GC_00001233§	MBARI-C16	2517702249	0.32	metabolism (polyamine)	agmatinase
GC_00001576	MBARI-C16	2517702141	0.48	metabolism (2^o^ met)	polyprenyl synthetase
GC_00001813	MBARI-C16	2517701728	0.49	metabolism (2^o^ met)	isoprenylcysteine carboxyl methyltransferase
GC_00002276§	MBARI-C16	2517702021	0.47	metabolism (sugar)	CDP-6-deoxy-D-xylo-4-hexulose-3-dehydrase
GC_00001670	MBARI-C16	2517701066	0.48	metabolism (sugar)	xylose isomerase domain protein TIM barrel
GC_00001567	MBARI-C16	2517701660	0.49	metabolism (sugar)	dTDP-4-amino-4,6-dideoxygalactose transaminase
GC_00000961†	MBARI-C16	2517702609	0.49	metabolism (sugar)	ribokinase
GC_00001926	MBARI-C16	2517700965	0.42	regulation	transcriptional regulator, LysR family
GC_00000837	MBARI-C16	2517702729	0.44	regulation	regulatory protein SoxS
GC_00000868	MBARI-C16	2517701134	0.45	regulation	transcriptional regulator, LysR family
GC_00001621	MBARI-C16	2517701264	0.46	regulation	transcriptional regulator, LysR family
GC_00000824	MBARI-C16	2236439675	0.42	regulation (pilin)	peptide-methionine (R)-S-oxide reductase
GC_00001628	MBARI-C16	2517702208	0.41	transcription	polyA polymerase
GC_00000610	MBARI-C16	2236438639	0.45	transport	ABC transporter permease protein
GC_00002178	MBARI-C16	Ga0315456_1082	0.38	transport (carboxylate)	TRAP transporter solute receptor TAXI family
GC_00001275	MBARI-C16	2517701302	0.44	unknown/other	hypothetical protein
GC_00001341	MBARI-C16	2517702560	0.47	unknown/other	selenium-binding protein

The expression ratio (transcripts per gene copy) is the mean across all sample dates. See Table S6 for annotation details. All are significantly different at *p* < 0.05 based on the Benjamini-Hochberg FDR adjustment. MBARI Clade, species with the higher gene expression; n/a, gene is from MAG not available in IMG; 2^o^ met = secondary metabolite. Genes with LGT signatures are indicated by *, Rhizobiaceae; †, Hyphomicrobiales; and §, Gammaproteobacteria.

### Differential gene content and expression

Our analysis focused on gene divergence potentially indicative of adaptive genetic variation by examining genes that were unique, unique laterally transferred (LGT), upregulated, or subjected to allelic replacement (*dS* outlier genes).

#### Carbon processing

**Fig. 4:**
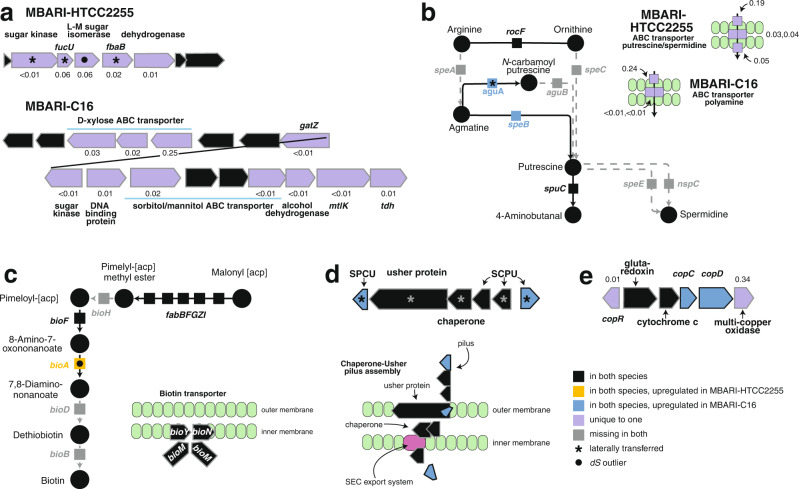
Gene composition and expression divergence between MBARI-HTCC2255 and MBARI-C16. **a** Sugar uptake and catabolism genes. *fucU*, L-fucose mutarotase; L-M, lyxose-mannose; *fbA*, fructose-bisphosphate aldolase; *gatZ*, tagatose-1,6-bisphosphate aldolase; *mtlK*, mannitol-2-dehydrogenase; *tdh*, threonine dehydrogenase. **b** Polyamine synthesis and uptake genes. *speA*, arginine carboxylase; *speB*, agmatinase; *speC*, ornithine decarboxylase; *speE*, spermidine synthase; *aguA*, agmatine deiminase; *aguB*, N-carbamoylputrescine amidase; *spuC*, putrescine-pyruvate transaminase; *nspC*, carboxynorspermidine decarboxylase. **c** Biotin synthesis and uptake genes. *bioA*, adenosylmethionine-8-amino-7-oxononanoate aminotransferase. **d** Chaperone-usher (CU) pilus synthesis genes. SCPU, spore coat U domain-containing protein. **e** Copper-related operon. *copR*, transcriptional regulator; *copC*, copper binding protein; *copD*, copper resistance protein. Expression levels are shown adjacent to each unique gene in units of transcripts per gene copy; the average expression level across all genes and all sample dates is 0.07 transcripts per gene copy.

Analysis of the species’ abilities to utilize carbon sources in the environment identified differential reliance on glycolytic sugars and polyamines. The MBARI-HTCC2255 genomes contained five co-located and unique genes with signals of lateral gene transfer, one of which (*fucU)* has been found to enhance the efficiency of glycolysis [64] (Table S2). Three components of a unique ABC carbohydrate transporter (substrate unknown) with *ds* signatures indicative of recent allelic replacement were also present in this species. In the genome of MBARI-C16, differential carbohydrate use was indicated by a 15-gene region encoding unique sugar transporters and catabolic genes suggestive of the ability to use a sugar alcohol (likely sorbitol or mannitol) and a pentulose (likely xylose) (Fig. 4a). Differences in gene content for polyamine uptake involved a unique transporter system in each species (Tables S2, S3).

#### Metabolic pathways

Organic sulfur and organic nitrogen utilization also had signatures of species’ differentiation. MBARI-HTCC2255 harbors a second degradation pathway for taurine (via *tauD*) and a second cleavage gene for marine osmolyte dimethylsulfoniopropionate (DMSP) (via *dddQ*). The MBARI-HTCC2255 genomes also have a unique choline dehydrogenase gene (Table S2) and higher expression further downstream in the choline pathway at sarcosine oxidation (Table 1). A biotin (vitamin B_7_) synthesis gene (*bioA*) was expressed at 4-fold higher levels in MBARI-HTCC2255 compared to -C16 (Fig. 4c, Table 1, S5) and MBARI-HTCC2255 also has a second copy of riboflavin biosynthesis gene *ribH* (Table S2).

#### Cell surface and antimicrobial features

Genes of the chaperone usher (CU) pilus system were significantly upregulated in MBARI-C16 (Table 1), suggesting a more important role in adhesion (potentially to other bacteria, host cells, or environmental surfaces). We hypothesized that this might indicate divergence between free-living versus surface-attached life histories. However, analysis of Tara Oceans Expedition metagenomic data [43] from stations with high NAC11-7 abundance did not support this hypothesis, as cells of both species were strongly biased toward free-living size fractions (Fig. S3). Genomic differences in antimicrobial capabilities were evident from a unique export system of a virulent lipoprotein in MBARI-HTCC2255 (Table S2), and two unique drug/metabolite permeases to export secondary metabolites from MBARI-C16. MBARI-HTCC2255 has a unique gene for polymerization of peptidoglycan, and MBARI-C16 has a unique gene altering cell surface polysaccharides (UDP-*N*-acetylglucosamine to UDP-*N*-acetylmannosamine) (Table S2), and a *d*_*s*_ outlier gene encoding synthesis of lipid A (Table S6).

#### Metals

A signal of greater reliance on copper was present in the MBARI-C16 genomes. Two unique genes mediating copper homeostasis and copper-catalyzed oxidation (Fig. 4e; Table S2), along with 16-fold and 20-fold upregulated copper export protein and copper-binding protein, differentiated the species (Figs. 3, 4e). Hints about the processes requiring a larger copper quota were difficult to glean, as the target and roles of the unique multi-copper oxidase is unclear [65]. However, four upregulated MBARI-C16 cytochrome-related genes are consistent with a greater copper demand for aerobic respiration (Table 1)

### Synthesis

Overall, there was good agreement among the gene categories of adaptive genetic variation with regard to functions that may underlie lineage divergence (Table S7). Two or more of the genes identified as unique (99 genes), upregulated (51 genes), recently replaced (11 genes), or laterally transferred (15 genes) supported differences in utilization of sugar and polyamine substrates (Fig. 4a,b), biotin synthesis (Fig. 4c), pilin synthesis (Fig. 4d), and copper quota (Fig. 4e, Table 1). These functional categories are consistent with a recent analysis of genes underlying speciation in a related marine Roseobacter lineage [66] that similarly pointed to differential utilization of sugars and polyamines, biotin acquisition, and metal acquisition as niche dimensions along which the species diverged.

From the in situ robotic collection and preservation of nucleic acid samples, internal standards allowed calculations of transcript numbers for all genes in the genomes on 27 dates in the same seawater environment. This type of information has rarely been available for evaluating the physiological or ecological differences that maintain bacterial species boundaries. In this case, 51 genes were upregulated in one or the other of the species under Monterey Bay Fall bloom conditions, amounting to 2% of core genes. However shifts observed in marine bacterial transcript pools linked to daily patterns in photosynthesis [18, 20, 21, 67] suggest that additional gene groups may be invoked through the diel cycle; if so, gene regulation plays a larger role in species differentiation than demonstrated by this mid-morning (10:00 am) dataset.

The similar overall pattern of the NAC11-7 species through the 7-week Monterey Bay bloom (Fig. 1b) suggested to us the possibility of complementary auxotrophies in the two species that were rescued via cross-feeding [68, 69]. Differences in gene content between the MBARI-HTCC2255 and MBARI-C16 genomes does not support this, however. Few of the genes unique to one species are assigned to essential pathways, and in those cases the gene functions are present in both species but the homologs differ (e.g., different shikimate dehydrogenases and 3-hydroxyacyl-CoA dehydrogenases). Monterey Bay metagenomic data collected two years prior to this study (Fall, 2014) [22] yielded an abundance ratio for the NAC11-7 species similar to that of Fall 2016; MBARI-HTCC2255:MBARI-C16 averaged 0.78:1 in 2014 and 0.85:1 in 2016. This was not the case, however, at three coastal Tara Oceans Expedition stations [43] with abundant NAC11-7 cells, where MBARI-HTCC2255 had much higher numbers (10:1 ratio), or at a Galapagos Island location [44] where only MBARI-C16 was present (Fig. S3). While the locations with high abundance of NAC11-7 cells were all coastal sites characterized by strong upwelling, the specific environmental factors that favored one species over the other are not known.

We noticed that the ratio of species abundance in Monterey Bay gradually shifted as the bloom progressed, providing a context in which to probe for environmental conditions that favored the success of MBARI-HTCC2255 at the earlier sample dates (MBARI-HTCC2255:MBARI-C16 abundance ratio of 1.30) and of MBARI-C16 during bloom demise (abundance ratio of 0.66). Measured abiotic variables of salinity, temperature, and mixed layer depth had no significant relationships with the MBARI-HTCC2255:MBARI-C16 abundance ratio. However, the biotic variables of chlorophyll *a* concentration, biomass of bloom dinoflagellate *Akashiwo sanguinea*, and dinoflagellate-derived metabolite dimethylsulfoniopropionate (DMSP) concentration were positively correlated with the ratio, such that both MBARI-HTCC2255 abundance and phytoplankton-related environmental parameters decreased through the sampling window relative to MBARI-C16 abundance (Fig. S4). These correlations offer a clue that MBARI-HTCC2255 cells have increased fitness over MBARI-C16 when phytoplankton biomass and activity are high. However, species divergence is likely influenced by the full history of the taxa and could be driven by greater fitness differences experienced in other habitats. In this coastal bloom ecosystem, co-occurring bacterial taxa with species-level ANI delineation diverged in 99 genes that represented 3% of the species’ pangenome, and in expression of 51 genes at mid-morning that represented 2% of the pangenome. For the two NAC11-7 species, the most strongly supported areas driving or maintaining differentiation were the utilization of carbohydrates and polyamines, features of the cell surface, and reliance on copper-catalyzed metabolism.

## Supplementary information

Supplemental Figures S1-S4

Supplemental Tables S1-S7

## Data Availability

Fall 2016 data are deposited at the National Center for Biotechnology Information’s Sequence Read Archive (SRA) under Umbrella Project PRJNA533622, which includes metagenomes (PRJNA467720 - PRJNA467773, PRJNA468208 - PRJNA468214, PRJNA502407 - PRJNA502427, PRJNA502440 - PRJNA502442), metatranscriptomes (PRJNA467774 - PRJNA467774, PRJNA468143 - PRJNA468143, PRJNA468299 - PRJNA468332, PRJNA502451 - PRJNA502468, PRJNA502608 - PRJNA502612), 16S rRNA gene amplicons (PRJNA511156 - PRJNA511206, PRJNA511216 - PRJNA511252), MAGs (PRJNA868720), and SAGs (PRJNA539227-PRJNA539307, PRJNA539368-PRJNA539478). Fall 2014 genomic data are deposited under PRJNA505827 (SAGs). Biological and chemical data are available at the Biological and Chemical Oceanography Data Management Office (BCO-DMO) under 10.1575/1912/bco-dmo.756376.1 and 10.1575/1912/bco-dmo.756413.1 [70, 71].
