# Supplementary material for: Ecological divergence of syntopic marine bacterial species is shaped by gene content and expression
Source: ISME J. 2023 Mar 4;17(6):813–22. doi: 10.1038/s41396-023-01390-4 (PMC10203303; doi:10.1038/s41396-023-01390-4)
Supplement: Supplementary file 1 — Supplemental Figures S1-S4 [file 41396_2023_1390_MOESM1_ESM.pdf]

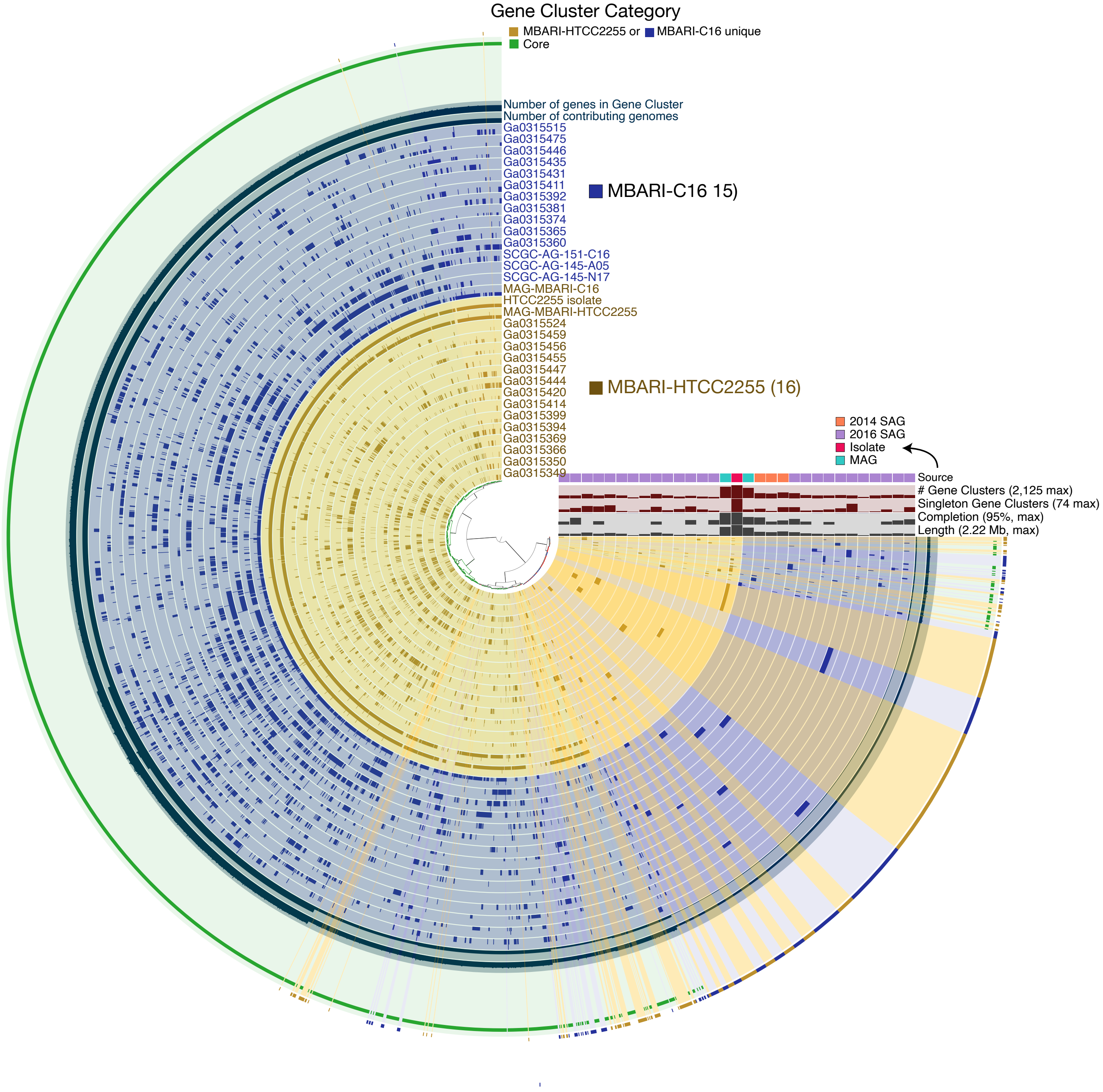

Figure S1. The MBARI HTCC2255:MBARI-C16 pangenome consists of 31 genomes, 19,250 genes, and 2,872 gene clusters. Bars in the first 31 rings (counting from the innermost) represent the occurrence of a gene cluster in a given genome. The next two rings describe the number of genomes contributing to the gene cluster and the total number of genes in the gene cluster. The outermost rings indicates whether gene cluster members are found in one clade or both (core genes).

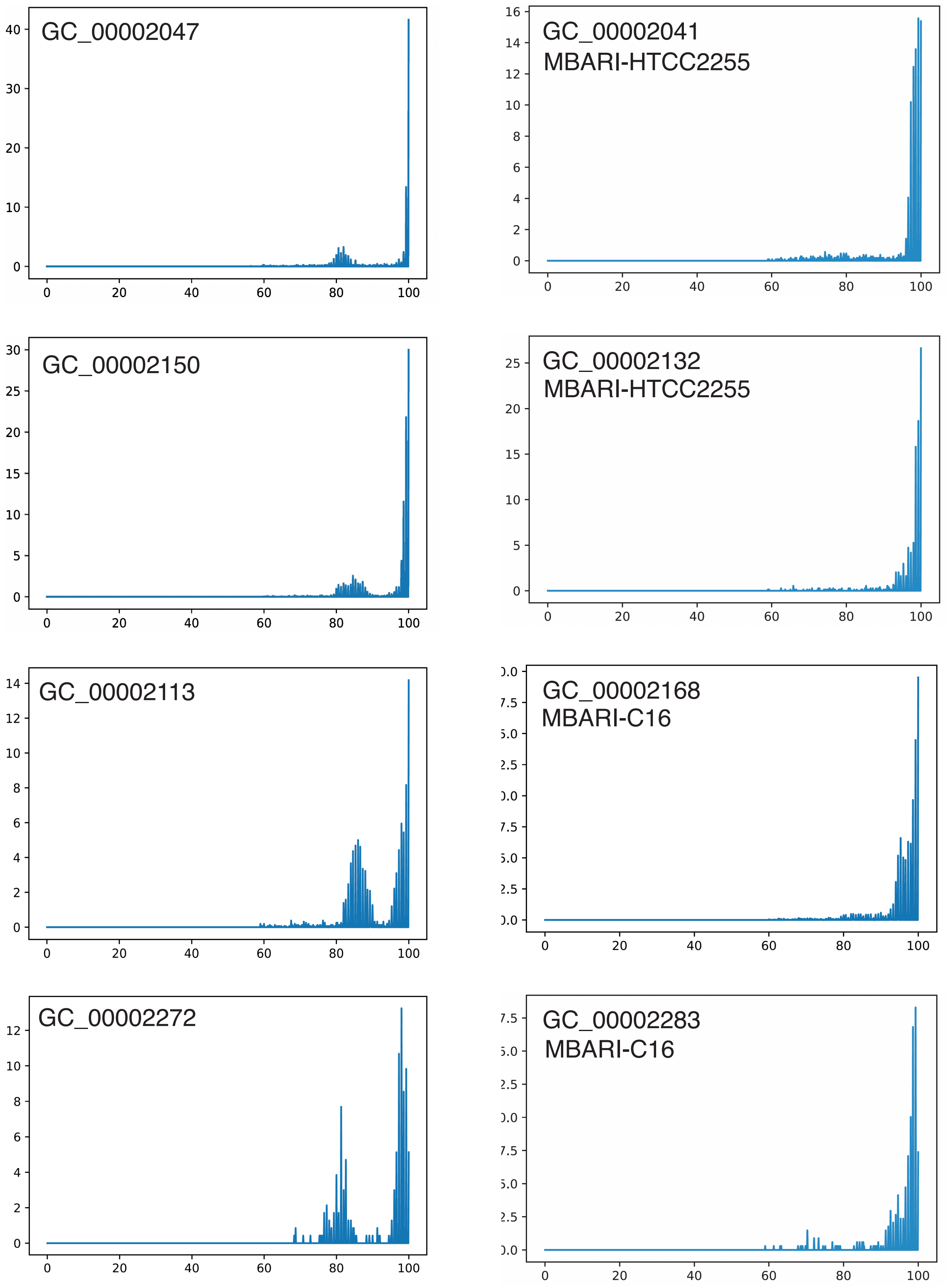

Figure S2. Confirmation step for candidate species-specific genes (those found multiple times in the 31 genomes but only in one species) based on mapping unassembled metagenomic reads. Plots on the left show genes with two identity peaks at ~100% and ~84% identity; these were reclassified to core (shared) genes. Plots on the right show genes with a single peak at ~100%; these were retained as unique genes.

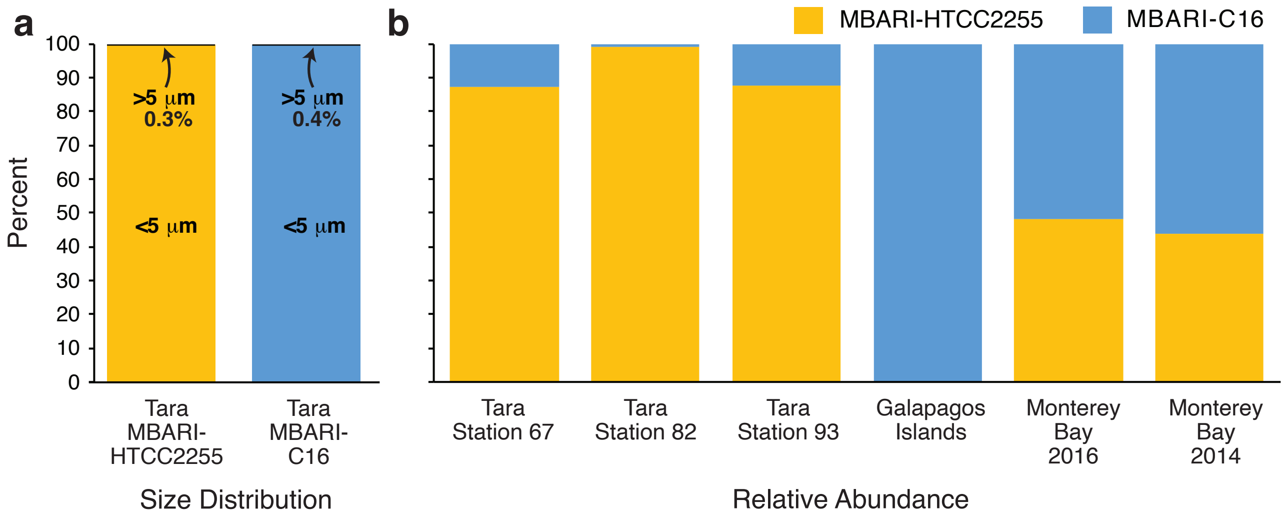

Figure S3. Abundance of MBARI-HTCC2255 and MBARI-C16 cells in surface ocean seawater. (a) Proportion of reads partitioning into the >5 μm size fraction (black) compared to the <5 μm size fraction (shaded), summed across three Tara Oceans Expedition samples. (b) Relative genome abundance determined from reads mapping to core functional genes of MBARI-HTCC2255 (gold shading) or MBARI-C16 (blue shading) in coastal upwelling samples from the Tara Oceans Expedition, Galapagos Islands, and Monterey Bay.

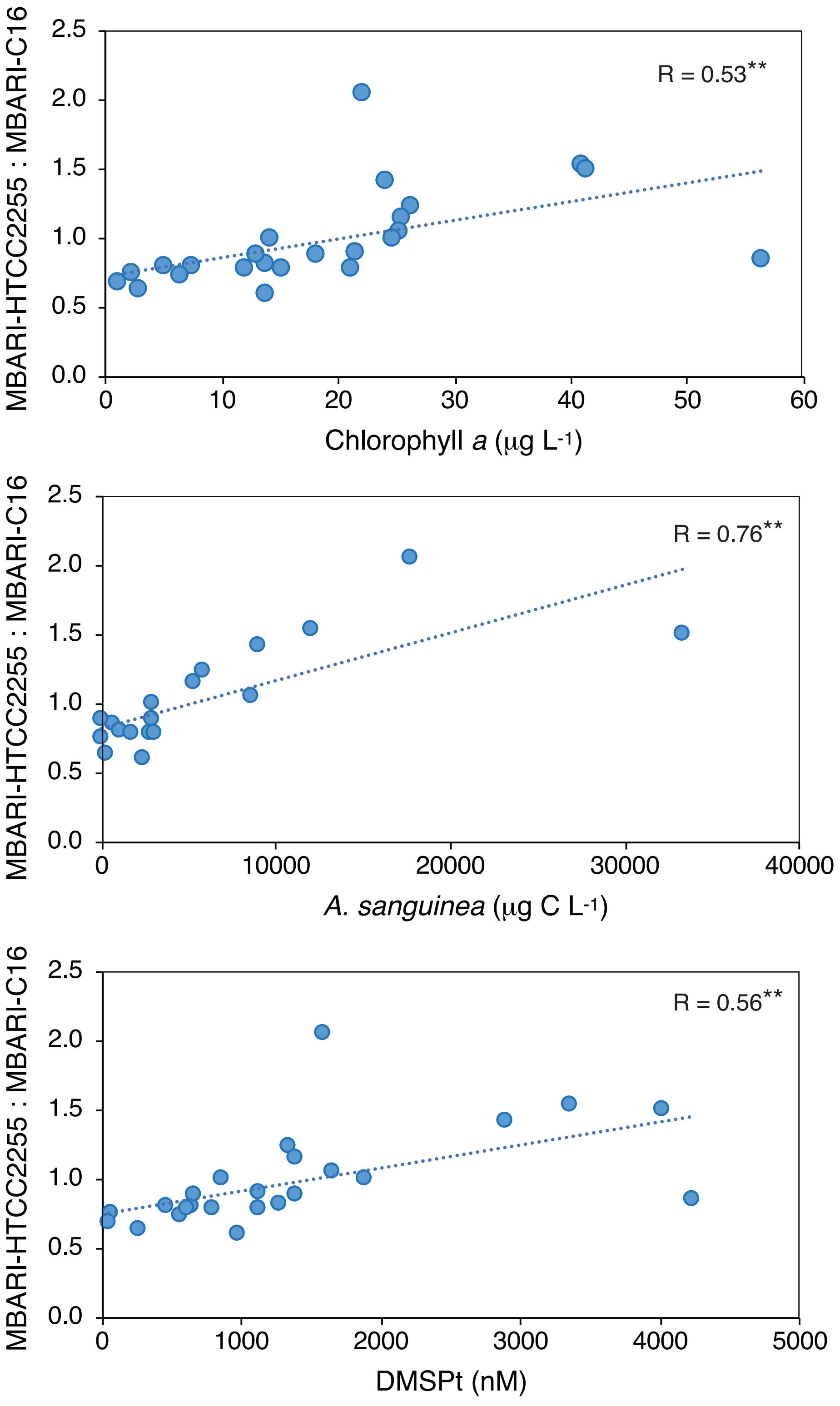

Figure S4. Correlations of the ratio of MBARI-HTCC2255:MBARI-C16 cell abundance to phytoplankton-related environmental parameters during the Fall 2016 Monterey Bay bloom. *A. sanguinea*, dinoflagellate species dominating the bloom; DMSPt, total dimethylsulfoniopropionate concentrations (total = particulate plus dissolved); **Correlation p value $\leq$ 0.01.
